# Design Method of High-Order Kalman Filter for Strong Nonlinear System Based on Kronecker Product Transform

**DOI:** 10.3390/s22020653

**Published:** 2022-01-15

**Authors:** Xiaohan Liu, Chenglin Wen, Xiaohui Sun

**Affiliations:** 1 School of Automation, Hangzhou Dianzi University, Hangzhou 310018, China; liuxiaohan@hdu.edu.cn (X.L.); sun_xh1993@hdu.edu.cn (X.S.); 2 School of Automation, Guangdong University of Petrochemical Technology, Maoming 525000, China

**Keywords:** Kronecker product, high-order Taylor expansion, Kalman filter, nonlinear system

## Abstract

In this paper, a novel design idea of high-order Kalman filter based on Kronecker product transform is proposed for a class of strong nonlinear stochastic dynamic systems. Firstly, those augmenting systems are modeled with help of the Kronecker product without system noise. Secondly, the augmented system errors are illustratively charactered by Gaussian white noise. Thirdly, at the expanded space a creative high-order Kalman filter is delicately designed, which consists of high-order Taylor expansion, introducing magical intermediate variables, representing linear systems converted from strongly nonlinear systems, designing Kalman filter, etc. The performance of the proposed filter will be much better than one of EKF, because it uses more information than EKF. Finally, its promise is verified through commonly used digital simulation examples.

## 1. Introduction

The problem of filtering has received extensive research and attention since it was put forward, and the design of filters has very important applications in the military industry, target tracking, and complex industrial systems [1,2,3,4,5,6]. In practical applications, due to the inherent and unexpected disturbances of the system, some adverse effects such as system oscillations or even loss of stability will occur. Therefore, the design of the filter becomes very important. First of all, for non-stationary systems, the most representative filter is the Kalman filter (KF) [7], which is an optimal filter for linear Gaussian systems derived from the least squares method based on the minimum variance.

In the actual background, almost all systems are nonlinear, so the filter design of nonlinear system has become the focus of research. In the case of weak nonlinearity, Extended Kalman Filter (EKF), which uses Taylor expansion to approximate the nonlinear system, was proposed in the literature [8]. Due to the better applicability of EKF, it is still widely studied and applied today. For example, the authors of [9] introduced the maximum correlation entropy and multi-dimensional Taylor net theory to the classic EKF algorithm, which overcomes the limitation that EKF can only be applied to Gaussian systems. In addition, in [10,11] the authors also optimized the EKF to improve the estimation accuracy of the filter and applied it in the direction of vehicle distance estimation and building structure damage detection. After EKF was first proposed to solve the problem of nonlinear Gaussian filtering, Unscented Kalman Filter (UFK), Cubature Kalman Filter (CKF), and Strong Tracking Filter (STF) were also proposed and further improved the estimation accuracy of the nonlinear Kalman filter [12,13,14]. After EKF was first proposed to solve the problem of nonlinear Gaussian filtering, UFK, CKF, and STF were also proposed and improved the estimation accuracy of the nonlinear Kalman filter. Furthermore, in [15] the advantages of adaptive fading UKF and robust UKF are combined to optimize the classic UKF, so that it has a good tracking ability even when the system model involves uncertainties. At the same time, the authors of [16] simplify the UKF and reduce the calculation process by applying the unscented transformation directly to the system model while ensuring the same performance; and the dual-use method and optimization of the Unscented Kalman Filter and Extended Kalman Filter are also proposed to improve the estimation quality in [17]. Recently, the estimation accuracy of STF has been further improved by extending it to a higher order extended form [18]; and a creative DLCKF with lower computational complexity and higher estimation accuracy than CKF has been proposed in [19]. Similarly, the application of Kalman filters in energy power systems and some cutting-edge directions has also been widely studied. In [20,21] the authors discussed the filter’s shock suppression function in eliminating energy systems; at the same time, the stability and reliability of lithium-ion battery SOC are improved by applying nonlinear Kalman filters in [22,23]; in addition, F. Mohamed also discussed the contribution of estimators and observers in the path recognition of mobile robots and nonlinear inverted pendulum viscous friction in [24,25].

Since the estimation accuracy of EKF is a first-order linear approximation, and the estimation accuracy of UKF and CKF are close to a second-order linear approximation [26,27,28]. However, as the degree of nonlinearity of the system increases, the rounding error and approximation error become larger and larger, making the result of the approximation worse, and even causing divergence. Therefore, carrying out research of nonlinear filtering methods in the case of strong nonlinearity and to consider the real nonlinear filtering problem has become the content of people’s subsequent consideration. Additionally, people have made breakthrough progress in this area. First, Polynomial Extended Kalman Filter (PEKF) was proposed in the literature [29,30,31], but it is difficult to understand; further, in [1] the authors introduce more difficult problems on the basis of the former. Their problem is that the expanded system introduces multiplicative noise terms and high-order noise terms, which not only increases the complexity of the system, but also makes the expanded system not conform to the standard Kalman filtering form; at the same time, PEKF and the literature [1] need to expand and merge the binomial when performing high-order expansion of the system, which further increases the computational complexity. Recently, the authors of [32] proposed a solution to the filtering problem of strong nonlinear systems. It defines higher-order terms as hidden variables and establishes a corresponding model, and then combines the hidden variables with the original variables to achieve system augmentation. Design and then state estimates are obtained through the proposed HEKF. However, there are also some problems in it. First, a type of additive typical case is given under special circumstances. The expression form is a special form, which is difficult to satisfy in general nonlinear systems. Secondly, it is necessary to carry out secondary modeling for the introduction of hidden variables, which increases the complexity of the problem. At the same time, the accuracy of secondary modeling needs to be further studied. Therefore, this thesis combines the two ideas and proposes a new method to solve the above two problems.

The core contribution of this paper is the design of a novel Kalman filter, the performance of which will be much better than one of EKF: (1) the modeling problem of the extended-dimensional model is solved theoretically by introducing the Kronecker product, which avoids the difficulty of finding prior information and the assumption of prior information caused by manual modeling; (2) system noise is not taken into account in the modeling of the system to simplify the modeling process, otherwise it will introduce a more difficult problem than the existing problem, that is, the system will create a product term between nonlinear noise term and nonlinear term; (3) a bridge from a nonlinear system to a linear system is erected, that is, the magical intermediate variable brought about by the higher-order Taylor expansion, which also avoids complicated calculations caused by polynomial expansion in the filtering process; (4) the complex problems are solved through the classic Kalman filter form, and the estimation accuracy is improved compared to EKF through the application of more high-order information; (5) compared with the modeling projection problem in the literature [29,30,31], the design process of the filter proposed in this paper is greatly simplified through the use of high-order information.

The remaining parts of this paper are organized as follows: in Section 2, the model of a type of nonlinear system targeted by the proposed filter is introduced; the method of expanding the dimensionality of the nonlinear system is given in Section 3; in Section 4, the design process of the proposed filter is given, and the algorithm of the filtering process is presented; Section 5 concerns simulation verification; Section 6 summarizes this article.

## 2. Problem Formulation

Consider a class of state and measurement models are discrete nonlinear dynamic stochastic systems:

The equation of the state of the system is

(1) $$u^{\left(1\right)}(\tau +1)=f^{\left(1\right)}(u^{\left(1\right)}(\tau ))+w^{\left(1\right)}(\tau )$$

The equation of the measurement of the system is

(2) $$z(\tau +1)=h(u^{\left(1\right)}(\tau +1))+v(\tau +1)$$

where, $u^{\left(1\right)}(\tau +1)={\left[\begin{array}{cccc} u_{1}^{\left(1\right)}(\tau +1) & u_{2}^{\left(1\right)}(\tau +1) & \cdots & u_{n}^{\left(1\right)}(\tau +1) \end{array}\right]}^{T}$ is the state vector to be estimated and $z(\tau +1)={\left[\begin{array}{cccc} z_{1}(\tau +1) & z_{2}(\tau +1) & \cdots & z_{u}(\tau +1) \end{array}\right]}^{T}$ is the observation; $w^{\left(1\right)}(\tau )={\left[\begin{array}{cccc} w_{1}^{\left(1\right)}(\tau ) & w_{2}^{\left(1\right)}(\tau ) & \cdots & w_{n}^{\left(1\right)}(\tau ) \end{array}\right]}^{T}$ and $v(\tau +1)={\left[\begin{array}{cccc} v_{1}(\tau +1) & v_{2}(\tau +1) & \cdots & v_{u}(\tau +1) \end{array}\right]}^{T}$ are the process and measurement noises; $f^{\left(1\right)}(u^{\left(1\right)}(\tau ))={\left[\begin{array}{cccc} f_{1}^{\left(1\right)}(u^{\left(1\right)}(\tau )) & f_{2}^{\left(1\right)}(u^{\left(1\right)}(\tau )) & \cdots & f_{n}^{\left(1\right)}(u^{\left(1\right)}(\tau )) \end{array}\right]}^{T}$ and $h(u^{\left(1\right)}(\tau +1))={\left[\begin{array}{cccc} h_{1}(u^{\left(1\right)}(\tau +1)) & h_{2}(u^{\left(1\right)}(\tau +1)) & \cdots & h_{u}(u^{\left(1\right)}(\tau +1)) \end{array}\right]}^{T}$ are the state mapping function and measurement mapping function of the system, respectively. The above variables and mapping functions satisfy the following assumptions. Additionally, these assumptions are considered as prerequisites for the filter design in this article.

**Assumption** **1.** *State noise* w(τ) *and measurement noise* v(τ+1) *are both independent random sequences.*

**Assumption** **2.** *State noise* w(τ)*and measurement noise*v(τ+1)*are both Gaussian white noise sequences, which meet the following conditions, respectively:* $E\{w^{\left(1\right)}(\tau )\}=0$*,*$E\{w^{\left(1\right)}(\tau ){\left(w^{\left(1\right)}(\tau )\right)}^{T}\}=Q^{\left(1\right)}(\tau )$*,*E{v(τ+1)}=0.

**Assumption** **3.** *The state transition function* f(u(τ))*has an* r+1*-order continuous partial derivative with respect to the state variable* u(τ)*, and the measurement function* h(u(τ+1))*has an* r+1*-order continuous partial derivative with respect to the state variable* u(τ+1)*.*

## 3. Augmented Modeling of Nonlinear Systems

In the filter designed in this paper, in order to avoid the introduction of multiplicative noise, which leads to an increase in system complexity and an increase in the difficulty of filter design, the system nonlinear function and system noise are modeled separately when the system is expanded. First, the sub-vector of the augmented system is designed, that is, the operation of the Kronecker product of order 1 is performed on the state transition function and the operation of the dimensional expansion of the nonlinear function is realized; after completing the calculation of the expansion of the nonlinear function, it is necessary to perform noise modeling on the expanded system model without noise. The method adopted in this paper aims to establish a nominal system and subtract the state transition functions of the nominal system and the noise-free augmented system to obtain a noise model of the corresponding dimension, thereby obtaining a complete augmented system. Specific steps are as follows.

**Definition** **1** ([1,30])**.** ${\left[u\right]}^{\left[l\right]}$ *represents the* l*-order Kronecker product of function* x*, which has the following properties*

(3) $${\left[u\right]}^{\left[l\right]}={\left[u\right]}^{\left[l-1\right]}⊗{\left[u\right]}^{\left[1\right]}$$

*where, the ‘* ⊗*’ is the symbol of the operation of the Kronecker product.*

*The state function described in Equation (1) is defined as follows.*

(4) $$u^{\left(l\right)}(\tau +1):={\left[u^{\left(1\right)}(\tau +1)\right]}^{\left[l\right]}$$

(5) $$f^{\left(l\right)}(u^{\left(1\right)}(\tau )):={\left[f^{\left(1\right)}(u^{\left(1\right)}(\tau ))\right]}^{\left[l\right]}$$

(6) $$w^{\left(l\right)}(\tau ):=u^{\left[l\right]}(\tau +1)-f^{\left(l\right)}(u^{\left(1\right)}(\tau ))$$

**Remark** **1.** *The system noise in Equation (6) is modeled by subtracting the nonlinear function term from the established nominal equation. This modeling method avoids the introduction of multiplicative noise and nonlinear noise terms.*

Therefore, we have a dynamic model of l-order hidden variables as

(7) $$u^{\left(l\right)}(\tau +1)=f^{\left(l\right)}(u^{\left(1\right)}(\tau ))+w^{\left(l\right)}(\tau )\;;\;l=2,3,\cdots ,r$$

where $w^{\left(l\right)}(\tau )$ is the modeling error of the l-order hidden variable $u^{\left(l\right)}(\tau +1)$, and it is assumed to satisfy $E\{w^{\left(l\right)}(\tau )\}=0$, $E\{w^{\left(l\right)}(\tau ){\left(w^{\left(l\right)}(\xi )\right)}^{T}\}=\delta_{\tau \xi }Q^{\left(l\right)}(\tau )$.

If the following form is noted

(8) $$U(\tau +1)={\left[\begin{array}{ccccc} {\left(u^{\left(1\right)}(\tau +1)\right)}^{T} & \cdots & {\left(u^{\left(l\right)}(\tau +1)\right)}^{T} & \cdots & {\left(u^{\left(r\right)}(\tau +1)\right)}^{T} \end{array}\right]}^{T}$$

(9) $$F(u^{\left(1\right)}(\tau ))={\left[\begin{array}{ccccc} {\left(f^{\left(1\right)}(u^{\left(1\right)}(\tau ))\right)}^{T} & \cdots & {\left(f^{\left(l\right)}(u^{\left(1\right)}(\tau ))\right)}^{T} & \cdots & {\left(f^{\left(r\right)}(u^{\left(1\right)}(\tau ))\right)}^{T} \end{array}\right]}^{T}$$

(10) $$W(\tau )={\left[\begin{array}{ccccc} {\left(w^{\left(1\right)}(\tau )\right)}^{T} & \cdots & {\left(w^{\left(l\right)}(\tau )\right)}^{T} & \cdots & {\left(w^{\left(r\right)}(\tau )\right)}^{T} \end{array}\right]}^{T}$$

where

(11) $$u^{\left(l\right)}(\tau +1)={\left[\begin{array}{ccccc} u_{1}^{\left(l\right)}(\tau +1) & \cdots & u_{i}^{\left(l\right)}(\tau +1) & \cdots & u_{n}^{\left(l\right)}(\tau +1) \end{array}\right]}^{T}$$

(12) $$f^{\left(l\right)}(u^{\left(1\right)}(\tau ))={\left[\begin{array}{ccccc} f_{1}^{\left(l\right)}(u^{\left(1\right)}(\tau )) & \cdots & f_{i}^{\left(l\right)}(u^{\left(1\right)}(\tau )) & \cdots & f_{n}^{\left(l\right)}(u^{\left(1\right)}(\tau )) \end{array}\right]}^{T}$$

(13) $$w^{\left(l\right)}(\tau )={\left[\begin{array}{ccccc} w_{1}^{\left(l\right)}(\tau ) & \cdots & w_{i}^{\left(l\right)}(\tau ) & \cdots & w_{n}^{\left(l\right)}(\tau ) \end{array}\right]}^{T}$$

The components in the above formula are, respectively,

(14) $$f_{i}^{\left(l\right)}(u^{\left(1\right)}(\tau ))=\prod_{\begin{array}{c} j=1\\ \sum_{i=1}^{n}l_{i}=l \end{array}}^{n}{\left(f_{j}^{\left(1\right)}(u^{\left(1\right)}(\tau ))\right)}^{l_{i}}\;;\;l=1,2,\cdots ,r;\;i=1,2,\cdots ,n_{l}$$

(15) $$w_{i}^{\left(l\right)}(\tau )=u_{i}^{\left[l\right]}(\tau +1)-f_{i}^{\left(l\right)}(u^{\left(1\right)}(\tau ))\;;\;l=1,2,\cdots ,r;\;i=1,2,\cdots ,n_{l}$$

(16) $$u_{i}^{\left(l\right)}(\tau +1)=f_{i}^{\left(l\right)}(u^{\left(1\right)}(\tau ))+w_{i}^{\left(l\right)}(\tau )\;;\;l=1,2,\cdots ,r;\;i=1,2,\cdots ,n_{l}$$

where $n_{l}$ is the number of hidden variables of order l, denoting $\left|\left\{\prod_{i=1}^{n}{\left(f_{i}^{\left(1\right)}(u^{\left(1\right)}(\tau ))\right)}^{l_{i}},\sum_{i=1}^{n}l_{i}=l\right\}\right|=n_{l}$.

Therefore, the augmented system can be as follows

(17) $$\begin{array}{l} U(\tau +1)=F(u^{\left(1\right)}(\tau ))+W(\tau )\\ z(\tau +1)=h(u^{\left(1\right)}(\tau +1))+v(\tau +1) \end{array}$$

where W(τ) is the modeling error of the augmented system, and E{W(τ)}=0, $E\{W(\tau ){\left(W(\xi )\right)}^{T}\}=Q(\tau )=diag\left\{\begin{array}{cccccc} Q^{\left(1\right)}(\tau ) & Q^{\left(2\right)}(\tau ) & \cdots & Q^{\left(l\right)}(\tau ) & \cdots & Q^{\left(r\right)}(\tau ) \end{array}\right\}$.

**Remark** **2.** *In this section, the augmented system is modeled by introducing the Kronecker product. This modeling method is theoretically deducible and interpretable.*

## 4. Filter Design

### 4.1. Filter Design

Since the filtering of nonlinear systems has been researched and promoted, various Kalman filters such as Extended Kalman filter (EKF), Unscented Kalman filter (UKF), and Cubature Kalman filter (CKF) have been proposed and researched. When the state model and the measurement model have sufficient accuracy, and the initial value of the filter is selected properly, the abovementioned nonlinear Kalman filter can give an accurate state estimated value. However, the usual situation is that the state and observation model usually have model uncertainty, that is, the model cannot completely match the nonlinear system described. The main reasons for this situation are as follows: the simplification of the model, the inaccuracy of the statistical characteristics of noise, the inaccurate modeling of the statistical characteristics of the initial state of the actual system, and the changes in the parameters of the actual system [1,33,34,35].

A very regrettable fact is that the Kalman filter has poor robustness with respect to model uncertainty, causing inaccurate state estimation and even divergence in the filtering process. Therefore, in order to solve this problem, in the proposed filter, the system state function is designed to expand the dimension to increase the system redundancy. Therefore, when the system state changes suddenly, the proposed filter also has better tracking ability.

When using a nonlinear Kalman filter for the filtering process, the main idea is to obtain an approximate linear system by approximating the nonlinear system. For example, EKF approximates a nonlinear system by using Taylor expansion to obtain the Jacobian matrix, while UKF is used to obtain the mean value of sampling points by random sampling near the approximate point. After the corresponding linearization system is obtained, the classical Kalman filter is used to filter it to obtain the state estimation value. The proposed filter design idea is similar to EKF, that is, the scalar of each sub-function of the extended system is subjected to the Taylor expansion of the selected order, and then stacked to obtain the linearized system and use the Kalman filter for filtering. The design steps of the filter are as follows, and the implementation of the filter algorithm proposed in this paper is shown in “Algorithm 1: Filtering process” at the end of this section.

Step 1: Time update.

Step 1.1: Calculate state prediction value.

(18) $$\hat{U}(\tau +1|\tau )=F({\hat{u}}^{\left(1\right)}(\tau |\tau ))$$

Step 1.2: Calculate the error of the state prediction.

(19) $$\begin{array}{l} \tilde{U}(\tau +1|\tau )=U(\tau +1)-\hat{U}(\tau +1|\tau )\\ =F(u^{\left(1\right)}(\tau ))-F({\hat{u}}^{\left(1\right)}(\tau |\tau ))+W(\tau )\\ =F({\hat{u}}^{\left(1\right)}(\tau |\tau ))+\sum_{l=1}^{r}A^{\left(l\right)}({\hat{u}}^{\left(1\right)}(\tau |\tau )){\tilde{u}}^{\left(l\right)}(\tau |\tau )+W(\tau )-F({\hat{u}}^{\left(1\right)}(\tau |\tau ))\\ =\sum_{l=1}^{r}A^{\left(l\right)}({\hat{u}}^{\left(1\right)}(\tau |\tau )){\tilde{u}}^{\left(l\right)}(\tau |\tau )+W(\tau ) \end{array}$$

**Remark** **3.** *The problem of increasing the computational complexity of the system due to the binomial expansion and merging introduced by the Taylor expansion is avoided here by modelingintermediate variables generated by high-order Taylor expansions corresponding to the order of Kronecker product expansions. See the (4.2) for the process of linearization of the nonlinear function* $F(u^{\left(1\right)}(\tau ))$*in Equation (19).*

Step 1.3: Find the covariance matrix of the state prediction error.

(20) $$\begin{array}{l} P(\tau +1|\tau )\\ =E\{\left[U(\tau +1)-\hat{U}(\tau +1|\tau )\right]{\left[U(\tau +1)-\hat{U}(\tau +1|\tau )\right]}^{T}\}\\ =E\{\left(\sum_{l=1}^{r}A^{\left(l\right)}({\hat{u}}^{\left(1\right)}(\tau |\tau )){\tilde{u}}^{\left(l\right)}(\tau |\tau )+W(\tau )\right){\left(\sum_{l=1}^{r}A^{\left(l\right)}({\hat{u}}^{\left(1\right)}(\tau |\tau )){\tilde{u}}^{\left(l\right)}(\tau |\tau )+W(\tau )\right)}^{T}\}\\ =\sum_{l=1}^{r}A^{\left(l\right)}({\hat{u}}^{\left(1\right)}(\tau |\tau ))P^{\left(l\right)}(\tau |\tau ){\left(A^{\left(l\right)}({\hat{u}}^{\left(1\right)}(\tau |\tau ))\right)}^{T}+Q(\tau ) \end{array}$$

Step 2: Measurement update.

Step 2.1: Calculation system measurement forecast.

(21) $$\hat{z}(\tau +1|\tau )=h({\hat{z}}^{\left(1\right)}(\tau +1|\tau ))$$

Step 2.2: Calculate system measurement prediction error.

(22) $$\begin{array}{l} \tilde{z}(\tau +1|\tau )=z(\tau +1)-\hat{z}(\tau +1|\tau )\\ =h(u^{\left(1\right)}(\tau +1))-h({\hat{u}}^{\left(1\right)}(\tau +1|\tau ))+v(\tau +1)\\ =h({\hat{u}}^{\left(1\right)}(\tau +1|\tau ))+\sum_{l=1}^{r}C^{\left(l\right)}({\hat{u}}^{\left(1\right)}(\tau +1|\tau )){\tilde{u}}^{\left(l\right)}(\tau +1|\tau )\\ +v(\tau +1)-h({\hat{u}}^{\left(1\right)}(\tau +1|\tau ))\\ =\sum_{l=1}^{r}C^{\left(l\right)}({\hat{u}}^{\left(1\right)}(\tau +1|\tau )){\tilde{u}}^{\left(l\right)}(\tau +1|\tau )+v(\tau +1) \end{array}$$

Step 2.3: Design Kalman filter.

(23) $$\hat{U}(\tau +1|\tau +1)=\hat{U}(\tau +1|\tau )+K(\tau +1)\tilde{z}(\tau +1|\tau )$$

Step 2.4: Solve for gain matrix K(τ+1).

First, give the form of the orthogonal decomposition of the measurement

(24) $$\begin{array}{l} z(\tau +1)=\hat{z}(\tau +1|\tau )+\tilde{z}(\tau +1|\tau )\\ =\hat{z}(\tau +1|\tau )+\sum_{l=1}^{r}C^{\left(l\right)}({\hat{u}}^{\left(1\right)}(\tau +1|\tau )){\tilde{u}}^{\left(l\right)}(\tau +1|\tau )+v(\tau +1) \end{array}$$

The estimated error

(25) $$\begin{array}{l} \tilde{U}(\tau +1|\tau +1)=U(\tau +1)-\hat{U}(\tau +1|\tau +1)\\ =U(\tau +1)-\hat{U}(\tau +1|\tau )-K(\tau +1)\tilde{z}(\tau +1|\tau )\\ =\tilde{U}(\tau +1|\tau )-K(\tau +1)\tilde{z}(\tau +1|\tau )\\ =\tilde{U}(\tau +1|\tau )-K(\tau +1)\left[\sum_{l=1}^{r}C^{\left(l\right)}({\hat{u}}^{\left(1\right)}(\tau +1|\tau )){\tilde{u}}^{\left(l\right)}(\tau +1|\tau )+v(\tau +1)\right] \end{array}$$

Utilize the Orthogonal Principle

(26) $$E\left\{\tilde{U}(\tau +1|\tau +1)z^{T}(\tau +1)\right\}=0$$

We have

(27) $$\begin{array}{l} E\left\{\tilde{U}(\tau +1|\tau +1)z^{T}(\tau +1)\right\}\\ =E\{\left[\tilde{U}(\tau +1|\tau )-K(\tau +1)\left(\sum_{l=1}^{r}C^{\left(l\right)}({\hat{u}}^{\left(1\right)}(\tau +1|\tau )){\tilde{u}}^{\left(l\right)}(\tau +1|\tau )+v(\tau +1)\right)\right]\\ {\left[h({\hat{u}}^{\left(1\right)}(\tau +1|\tau ))+\sum_{l=1}^{r}C^{\left(l\right)}({\hat{u}}^{\left(1\right)}(\tau +1|\tau ))\times {\tilde{u}}^{\left(l\right)}(\tau +1|\tau )+v(\tau +1)\right]}^{T}\}\\ =P(\tau +1|\tau )C^{T}({\hat{u}}^{\left(1\right)}(\tau +1|\tau ))-K(\tau +1)R(\tau +1)\\ -K(\tau +1)\sum_{l=1}^{r}C^{\left(l\right)}({\hat{u}}^{\left(1\right)}(\tau +1|\tau ))P^{\left(l\right)}(\tau +1|\tau ){\left(C^{\left(l\right)}({\hat{u}}^{\left(1\right)}(\tau +1|\tau ))\right)}^{T}=0 \end{array}$$

Therefore, we have

(28) $$\begin{array}{l} K(\tau +1)=P(\tau +1|\tau )C^{T}({\hat{u}}^{\left(1\right)}(\tau +1|\tau ))[\sum_{l=1}^{r}C^{\left(l\right)}({\hat{u}}^{\left(1\right)}(\tau +1|\tau ))\\ {P^{\left(l\right)}(\tau +1|\tau ){\left(C^{\left(l\right)}({\hat{u}}^{\left(1\right)}(\tau +1|\tau ))\right)}^{T}+R(\tau +1)]}^{-1} \end{array}$$

Step 2.5: Calculate the estimated error covariance matrix.

(29) $$\begin{array}{l} P(\tau +1|\tau +1)\\ =E\{\left[\tilde{U}(\tau +1|\tau +1)\right]{\left[\tilde{U}(\tau +1|\tau +1)\right]}^{T}\}\\ =P(\tau +1|\tau )-P(\tau +1|\tau )C^{T}({\hat{u}}^{\left(1\right)}(\tau +1|\tau ))K^{T}(\tau +1)-K(\tau +1)C({\hat{u}}^{\left(1\right)}(\tau +1|\tau ))P(\tau +1|\tau )\\ +K(\tau +1)(\sum_{l=1}^{r}C^{\left(l\right)}({\hat{u}}^{\left(1\right)}(\tau +1|\tau ))P^{\left(l\right)}(\tau +1|\tau ){\left(C^{\left(l\right)}({\hat{u}}^{\left(1\right)}(\tau +1|\tau ))\right)}^{T})K^{T}(\tau +1)+K(\tau +1)\\ \times R(\tau +1)K^{T}(\tau +1) \end{array}$$

Substituting Equation (27) into Equation (29), we get

(30) $$P(\tau +1|\tau +1)=\left[I-K(\tau +1)C({\hat{u}}^{\left(1\right)}(\tau +1|\tau ))\right]P(\tau +1|\tau )$$

At this point, the design of the filter is completed, and simulation tests are carried out in Section 5 to verify the effectiveness of the proposed filter.

### 4.2. Linearization of Nonlinear Functions

The design process of the filter is given above. In order to ensure the complete derivation of the filter formula, the linearization process of the stacked high-order nonlinear terms is given here. The higher-order Taylor expansion of the same order as the state expansion order is used to ensure that the higher-order variables on both sides of the expanded equation correspond to each other, which further improves the estimation accuracy of the filter. Specific steps are as follows:

From Equation (18), we can see

(31) $$\tilde{U}(\tau +1|\tau )=F(u^{\left(1\right)}(\tau ))-F({\hat{u}}^{\left(1\right)}(\tau |\tau ))+W(\tau )$$

From the third section, we have

(32) $$F(u^{\left(1\right)}(\tau ))={\left[\begin{array}{ccccc} {\left(f^{\left(1\right)}(u^{\left(1\right)}(\tau ))\right)}^{T} & \cdots & {\left(f^{\left(l\right)}(u^{\left(1\right)}(\tau ))\right)}^{T} & \cdots & {\left(f^{\left(r\right)}(u^{\left(1\right)}(\tau ))\right)}^{T} \end{array}\right]}^{T}$$

where

(33) $$f^{\left(l\right)}(u^{\left(1\right)}(\tau ))={\left[\begin{array}{ccccc} f_{1}^{\left(l\right)}(u^{\left(1\right)}(\tau )) & \cdots & f_{i}^{\left(l\right)}(u^{\left(1\right)}(\tau )) & \cdots & f_{n}^{\left(l\right)}(u^{\left(1\right)}(\tau )) \end{array}\right]}^{T}$$

For $f_{i}^{\left(l\right)}(u^{\left(1\right)}(\tau ))$, we have

(34) $$f_{i}^{\left(l\right)}(\tau +1)=\prod_{\begin{array}{c} j=1\\ \sum_{i=1}^{n}l_{i}=l \end{array}}^{n}{\left(f_{j}^{\left(1\right)}(u^{\left(1\right)}(\tau ))\right)}^{l_{i}}\;;l=1,2,\cdots ,r;\;i=1,2,\cdots ,n_{l}$$

Perform r-order Taylor expansion of $f_{i}^{\left(l\right)}(u^{\left(1\right)}(\tau ))$ at $u_{i}^{\left(1\right)}(\tau )={\hat{u}}_{i}^{\left(1\right)}(\tau |\tau )$ to get

(35) $$f_{i}^{\left(l\right)}(u^{\left(1\right)}(\tau ))=f_{i}^{\left(l\right)}({\hat{u}}^{\left(1\right)}(\tau |\tau ))+\sum_{l=1}^{r}\left\{\sum_{l_{1}+\cdots +l_{n}=l}a_{i;l_{1}l_{2}\cdots l_{n}}^{\left(l\right)}\prod_{\begin{array}{c} i=1\\ l_{1}+\cdots +l_{n}=l \end{array}}^{n}{\left[u_{i}^{\left(1\right)}(\tau )-{\hat{u}}_{i}^{\left(1\right)}(\tau |\tau )\right]}^{l_{i}}\right\}$$

where the $a_{i;l_{1}l_{2}\cdots l_{n}}^{\left(l\right)}$ is the coefficients of the above formula for Taylor expansion. Let ${\tilde{u}}_{i}^{\left(1\right)}(\tau ):=u_{i}^{\left(1\right)}(\tau )-{\hat{u}}_{i}^{\left(1\right)}(\tau |\tau )$, therefore, the above formula can be written as

(36) $$f_{i}^{\left(l\right)}(u^{\left(1\right)}(\tau ))=f_{i}^{\left(l\right)}({\hat{u}}^{\left(1\right)}(\tau |\tau ))+\sum_{l=1}^{r}\left\{\sum_{l_{1}+\cdots +l_{n}=l}a_{i;l_{1}l_{2}\cdots l_{n}}^{\left(l\right)}\prod_{\begin{array}{c} i=1\\ l_{1}+\cdots +l_{n}=l \end{array}}^{n}{\left[{\tilde{u}}_{i}^{\left(1\right)}(\tau )\right]}^{l_{i}}\right\}$$

We have

(37) $$\begin{array}{l} f^{\left(l\right)}(\tau +1)=\left[\begin{array}{c} \sum_{l=1}^{r}\left\{\sum_{l_{1}+\cdots +l_{n}=l}a_{1;l_{1}l_{2}\cdots l_{n}}^{\left(l\right)}\prod_{\begin{array}{c} i=1\\ l_{1}+\cdots +l_{n}=l \end{array}}^{n}{\left[{\tilde{u}}_{i}^{\left(1\right)}(\tau )\right]}^{l_{i}}\right\}\\ \vdots \\ \sum_{l=1}^{r}\left\{\sum_{l_{1}+\cdots +l_{n}=l}a_{i;l_{1}l_{2}\cdots l_{n}}^{\left(l\right)}\prod_{\begin{array}{c} i=1\\ l_{1}+\cdots +l_{n}=l \end{array}}^{n}{\left[{\tilde{u}}_{i}^{\left(1\right)}(\tau )\right]}^{l_{i}}\right\}\\ \vdots \\ \sum_{l=1}^{r}\left\{\sum_{l_{1}+\cdots +l_{n}=l}a_{n_{l};l_{1}l_{2}\cdots l_{n}}^{\left(l\right)}\prod_{\begin{array}{c} i=1\\ l_{1}+\cdots +l_{n}=l \end{array}}^{n}{\left[{\tilde{u}}_{i}^{\left(1\right)}(\tau )\right]}^{l_{i}}\right\} \end{array}\right]+\left[\begin{array}{c} f_{1}^{\left(l\right)}({\hat{u}}^{\left(1\right)}(\tau |\tau ))\\ \vdots \\ f_{i}^{\left(l\right)}({\hat{u}}^{\left(1\right)}(\tau |\tau ))\\ \vdots \\ f_{n_{l}}^{\left(l\right)}({\hat{u}}^{\left(1\right)}(\tau |\tau )) \end{array}\right]\\ =\left[\begin{array}{ccc} a_{1;l_{1}}^{\left(l\right)} & \cdots & a_{1;n_{l}}^{\left(l\right)}\\ \vdots & \begin{array}{ccc} \ddots & & \\ & a_{i;l_{1}l_{2}\cdots l_{n}}^{\left(l\right)} & \\ & & \ddots \end{array} & \vdots \\ a_{n_{l};1}^{\left(l\right)} & \cdots & a_{n_{l};n_{l}}^{\left(l\right)} \end{array}\right]\left[\begin{array}{c} {\left[{\tilde{u}}_{1}^{\left(1\right)}(\tau )\right]}^{l}\\ \vdots \\ \prod_{\begin{array}{c} i=1\\ l_{1}+\cdots +l_{n}=l \end{array}}^{n}{\left[{\tilde{u}}_{i}^{\left(1\right)}(\tau )\right]}^{l_{i}}\\ \vdots \\ {\left[{\tilde{u}}_{n}^{\left(1\right)}(\tau )\right]}^{l} \end{array}\right]+\left[\begin{array}{c} f_{1}^{\left(l\right)}({\hat{u}}^{\left(1\right)}(\tau |\tau ))\\ \vdots \\ f_{i}^{\left(l\right)}({\hat{u}}^{\left(1\right)}(\tau |\tau ))\\ \vdots \\ f_{n_{l}}^{\left(l\right)}({\hat{u}}^{\left(1\right)}(\tau |\tau )) \end{array}\right]\\ =A^{\left(l\right)}({\hat{x}}^{\left(l\right)}(\tau |\tau )){\tilde{u}}^{\left(l\right)}(\tau )+f^{\left(l\right)}({\hat{u}}^{\left(1\right)}(\tau |\tau )) \end{array}$$

Finally, each sub-function in $F(u^{\left(1\right)}(\tau ))$ is subjected to the above linearization process separately to obtain

(38) $$F(u^{\left(1\right)}(\tau ))=F({\hat{u}}^{\left(1\right)}(\tau |\tau ))+\sum_{l=1}^{r}A^{\left(l\right)}({\hat{u}}^{\left(1\right)}(\tau |\tau )){\tilde{u}}^{\left(l\right)}(\tau |\tau )$$

Similarly, the other nonlinear terms can also be used in this way to achieve their linearization process.
**Algorithm 1:** Filtering process.Step 1.Model the augmented form of the state equation based on (8)–(16)Step 2.Realize the linearization process of the nonlinear system to obtain $A^{\left(l\right)}({\hat{u}}^{\left(1\right)}(\tau |\tau ))$ and linear equation based on (35)–(38)Step 3.Calculate state prediction value $\hat{U}(\tau +1|\tau )$ according to (18)Step 4.Calculate the error of the state prediction $\tilde{U}(\tau +1|\tau )$ according to (19)Step 5.Determine the covariance matrix of the state prediction error P(τ+1|τ) with (20)Step 6.Compute the forecast and prediction error of system measurement $\hat{z}(\tau +1|\tau )$ and $\tilde{z}(\tau +1|\tau )$ with (21) and (22)Step 7.Solve for Kalman gain matrix K(τ+1) based on (24)–(28)Step 8.Calculate the state estimate $\hat{U}(\tau +1|\tau +1)$ at τ+1 using (23)Step 9.Update the estimated error covariance matrix by (28)–(30)Step 10.Set τ=τ+1 and go to Step 2.

**Remark** **4.** *The filter designed in this section extends the classic Kalman filter to the augmented system designed above and simplifies the computational complexity by establishing magical intermediate variables in (36). At the same time, the filter designed in this paper also avoids the projection problem introduced in [1], and further simplifies the filter design process.*

## 5. Numerical Simulations

In this section, two cases in the literature [1,30] are used to illustrate the effectiveness of the proposed method; at the same time, the classic EKF is also used to filter them, and the performance of the algorithm is proved by comparison. In the realization of the simulation process of the following two cases, a total of 50 iterations of 1–50 times are carried out. Additionally, EKF, the proposed filter expanded to the second order and the proposed filter expanded to the third order are, respectively, used to obtain the corresponding estimated values.

### 5.1. Case 1

**Figure 1:**
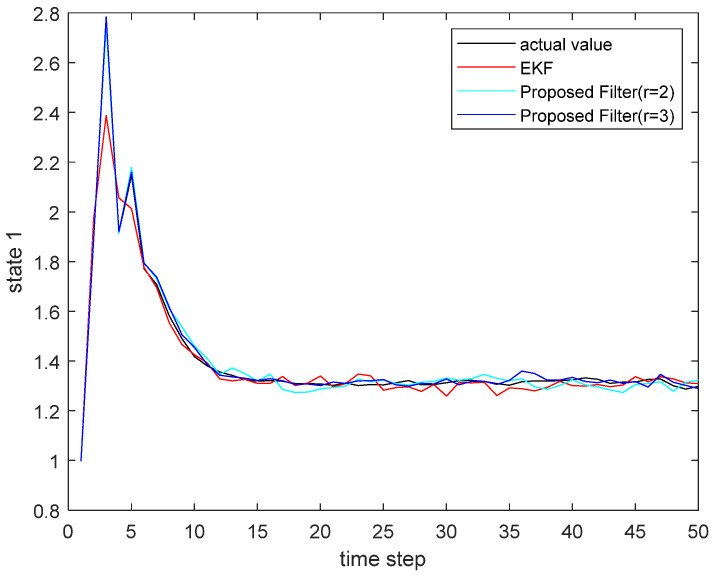
The actual state $u_{1}$ and its estimate.

**Figure 2:**
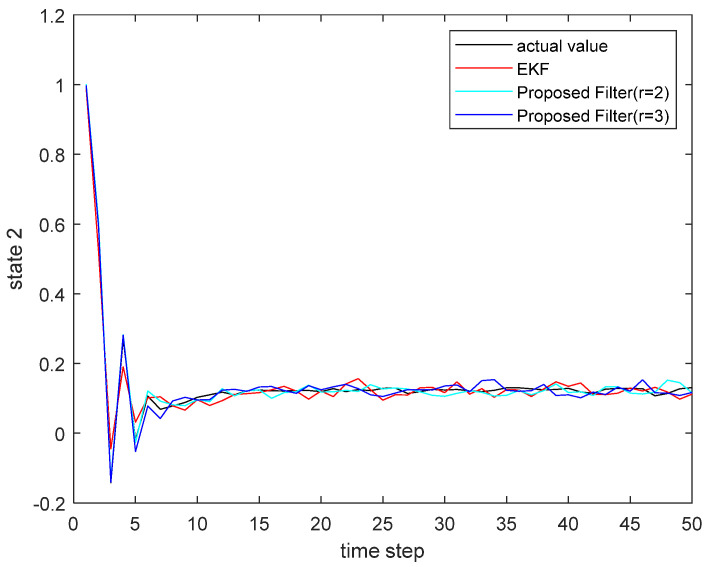
The actual state $u_{2}$ and its estimate.

**Figure 3:**
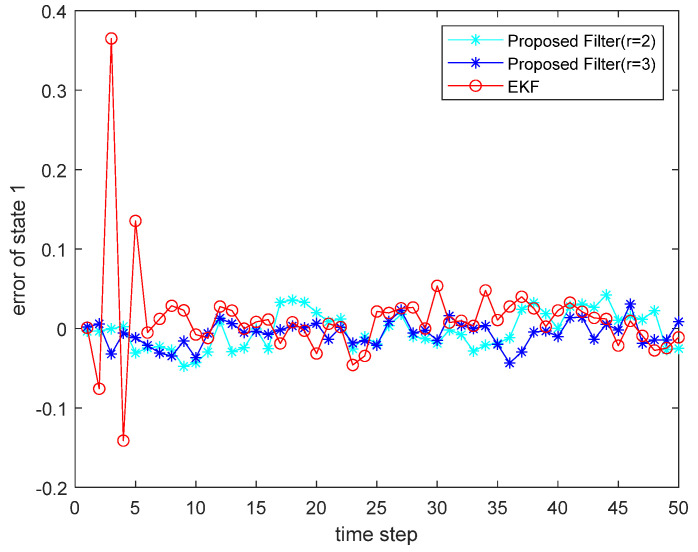
Estimated error for state $u_{1}$.

**Figure 4:**
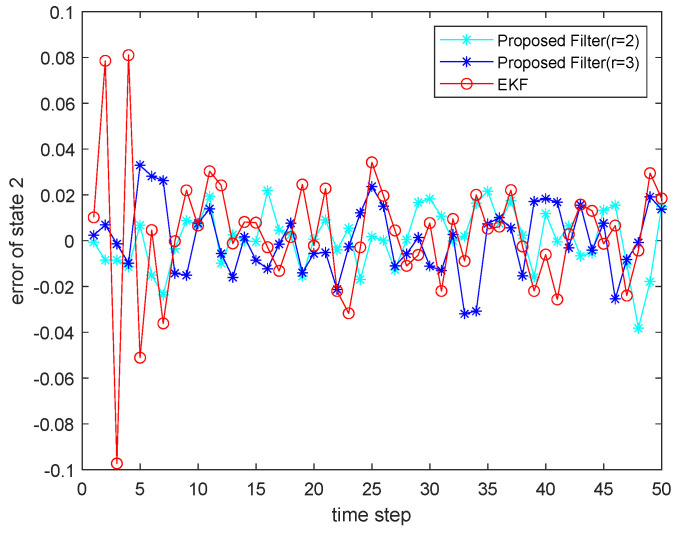
Estimated error for state $u_{1}$.

Consider the following nonlinear discrete system [30]:

$$\left\{ \begin{array}{l} u_{1}(\tau +1)=0.8u_{1}(\tau )+u_{1}(\tau )u_{2}(\tau )+0.1+0.01w_{1}(\tau )\\ u_{2}(\tau +1)=1.5u_{2}(\tau )-u_{1}(\tau )u_{2}(\tau )+0.1+0.01w_{2}(\tau ) \end{array}\right.$$

$$\left\{ \begin{array}{l} z_{1}(\tau +1)=u_{1}(\tau +1)+0.04v_{1}(\tau +1)\\ z_{2}(\tau +1)=u_{2}(\tau +1)+0.04v_{2}(\tau +1) \end{array}\right.$$

where the state noise and the measurement noise are both uncorrelated white Gaussian noise and obey the following distribution: w(τ)∼N(0,Q), v(τ)∼N(0,R), Q=diag(0.1,0.2), R=diag(0.1,0.2); the initial values of the system are $x(0)={\left[1,1\right]}^{T}$, $P(0|0)=I_{2\times 2}$. The simulation results are shown in the following figure. Figure 1 and Figure 2 show the output curves of the true value of state $u_{1}$ and state $u_{2}$, the estimated value of EKF, and the estimated value when the proposed filter is extended to the second and third orders; Figure 3 and Figure 4 show the estimated error output results of the above filter in state $u_{1}$ and state $u_{2}$, respectively; Table 1 shows the comparison of the mean square error of each filtering method after the system is stabilized.

### 5.2. Case 2

**Figure 5:**
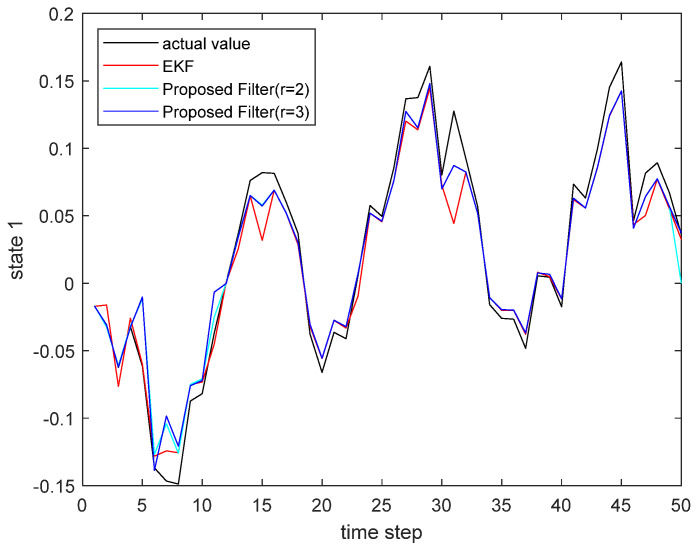
The actual state $u_{1}$ and its estimate.

**Figure 6:**
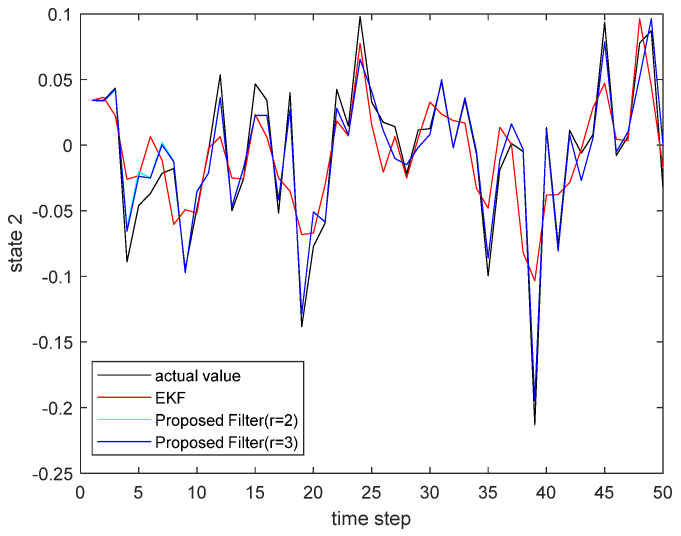
The actual state $u_{2}$ and its estimate.

**Figure 7:**
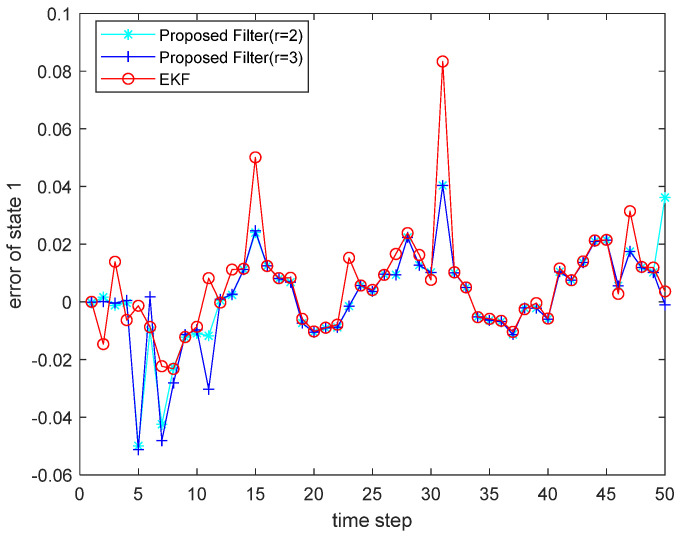
Estimated error for state $u_{1}$.

**Figure 8:**
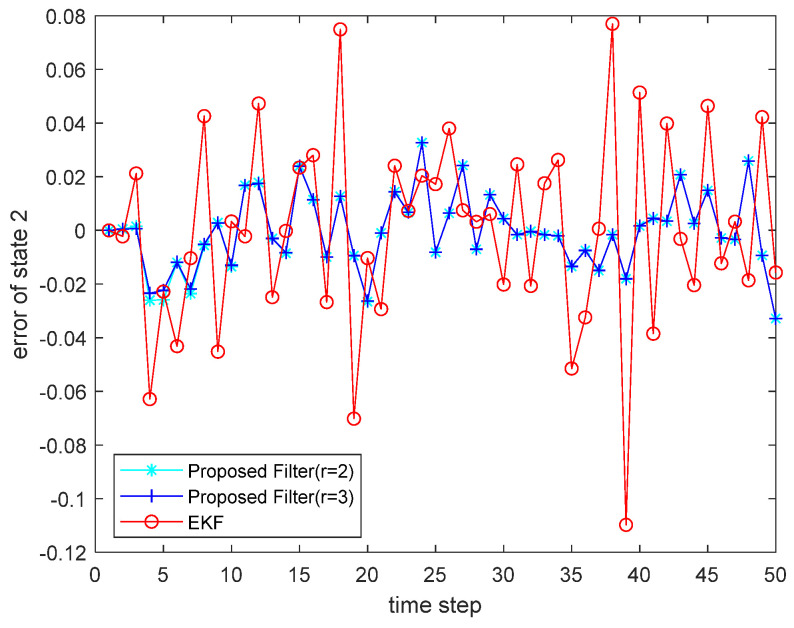
Estimated error for state $u_{1}$.

Consider the following nonlinear discrete system [1]:

$$\left\{ \begin{array}{l} u_{1}(\tau +1)=0.85u_{1}(\tau )+0.5u_{2}(\tau )\sin(u_{1}(\tau ))+w_{1}(\tau )+\alpha (\tau )\\ u_{2}(\tau +1)=1.15\beta (\tau )-0.5u_{1}(\tau )\sin(u_{2}(\tau ))+w_{2}(\tau )\\ \;\;z(\tau +1)=x_{2}(\tau +1)+v(\tau +1)\\ \;\;\beta (\tau )=z(\tau ) \end{array}\right.$$

where the α(τ) is

$$\alpha (\tau )=\left\{ \begin{array}{ll} 0.003, & \;\mathrm{if}\;\tau >20\\ 0, & \;\mathrm{otherwise} \end{array}\right.$$

where the state noise and the measurement noise are both uncorrelated white Gaussian noise and obey the following distribution: w(τ)∼N(0,Q), v(τ)∼N(0,R), Q=diag(0.002,0.002), R=0.004; the initial values of the system are $u(0)={\left[0,0\right]}^{T}$, $P(0|0)=I_{2\times 2}$. The simulation results are shown in the following figure. Figure 5 and Figure 6 present the output curves of the true value of state $u_{1}$ and state $u_{2}$, the estimated value of EKF, and the estimated value when the proposed filter is extended to the second and third orders; Figure 7 and Figure 8 show the estimated error output results of the above filter in state $u_{1}$ and state $u_{2}$, respectively; Table 2 shows the comparison of the mean square error of each filtering method after the system is stabilized.

### 5.3. Summary of Simulation Results

In case 1, compared with EKF, the estimation accuracy of the proposed filter is increased by 21.50% when it is extended to the second order, and when the proposed filter is extended to the third order, the estimation accuracy is increased to 48.31% relative to EKF. For case 2, compared with EKF, the estimation accuracy of the proposed filter is improved by 73.09% when it is extended to the second order, and when the proposed filter is extended to the third order, the estimation accuracy is improved to 75.70% relative to EKF. According to the above results, it can be seen that the filter proposed has a significant improvement in estimation accuracy compared to EKF, and as the expansion order increases, the estimation accuracy also increases. At the same time, when the system state has a sudden change, the proposed filter also has a good tracking ability.

## 6. Conclusions and Future

This paper studied the design of the augmented model and the novel high-order Kalman filter for a class of nonlinear Gaussian systems. Compared with the classic extended Kalman filter and unscented Kalman filter, the filter proposed in this paper used more high-order information to achieve better estimation accuracy and tracking ability. First of all, the augmented system was modeled by introducing the Kronecker product and performing the Kronecker product operation of the selected order on the nonlinear term of the system state function. In addition, the process of dimensional expansion was simplified by ignoring the system noise when modeling the augmented system, which also avoided the introduction of product term between nonlinear noise term and nonlinear term. Then, the classic Kalman filter was extended to this augmented system by introducing creative intermediate variables brought by high-order Taylor expansion, which reduced the difficulty of filter design and the complexity of calculations. Through the comparison of simulation, it can be found that when the system state changed suddenly, the proposed method had better tracking performance, and the estimated errors were also significantly reduced. In addition, as the expansion order of the system increases, the estimated accuracy of the proposed filter also increases. Such results prove that the proposed filter has better robustness and estimation accuracy.

There are still several points worthy of improvement in future research. One of the most important points is that the system state noise is not taken into consideration in the augmented design of the system in this paper, which makes the proposed augmented system an incompletely extended form. Therefore, we will consider the system state noise into the dimensional expansion operation of system to realize a complete augmented system and complete the design of the corresponding filter, which will further increase the performance of the filter.

## Figures and Tables

**Table 1:** Error comparison between Proposed Filter and EKF in case 1.

		EKF	Proposed Filter (r = 2)	Proposed Filter (r = 3)
MSE of $u_{1}$		$5.58\times 10^{-4}$	$4.78\times 10^{-4}$	$2.25\times 10^{-4}$
MSE of $u_{2}$		$2.69\times 10^{-4}$	$1.72\times 10^{-4}$	$2.03\times 10^{-4}$
MSE of u		$4.14\times 10^{-4}$	$3.25\times 10^{-4}$	$2.14\times 10^{-4}$
Improved (%)	$u_{1}$	×	14.34%	52.93%
	$u_{2}$	×	39.06%	−18.02%
Improved of u (%)		×	21.50%	34.15%
Improved relative to EKF (%)	$u_{1}$	×	14.34%	59.68%
	$u_{2}$	×	39.06%	24.54%
Improved of u (%)		×	21.50%	48.31%

**Table 2:** Error comparison between Proposed Filter and EKF in case 2.

		EKF	Proposed Filter (r = 2)	Proposed Filter (r = 3)
MSE of $u_{1}$		$2.34\times 10^{-4}$	$1.28\times 10^{-4}$	$1.02\times 10^{-4}$
MSE of $u_{2}$		$7.76\times 10^{-4}$	$1.26\times 10^{-4}$	$1.26\times 10^{-4}$
MSE of u		$5.05\times 10^{-4}$	$1.27\times 10^{-4}$	$1.14\times 10^{-4}$
Improved (%)	$u_{1}$	×	45.30%	20.31%
	$u_{2}$	×	83.76%	0%
Improved of u (%)		×	74.85%	10.24%
Improved relative to EKF (%)	$u_{1}$	×	45.30%	59.41%
	$u_{2}$	×	83.76%	83.76%
Improved of u (%)		×	74.85%	77.43%

## Nomenclature

u	state vector
z	observation vector
w	process noise
v	measurement noise
f(·)	nonlinear state function
h(·)	nonlinear measurement function
τ	time step
$\tilde{u}$	intermediate variable
$\hat{u}(\tau |\tau )$	state estimate
$\hat{u}(\tau +1|\tau )$	state prediction value
$\hat{z}(\tau +1|\tau )$	measurement prediction value
P(τ+1|τ)	state prediction error covariance matrix
P(τ+1|τ+1)	estimate error covariance matrix
K(τ+1)	Kalman gain matrix
